# Li–Fraumeni syndrome in Tunisian carriers with different and rare tumor phenotype: genotype–phenotype correlation

**DOI:** 10.1186/s12920-022-01189-w

**Published:** 2022-03-04

**Authors:** Hela Sassi, Rym Meddeb, Mohamed Aziz Cherif, Chiraz Nasr, Aouatef Riahi, Samia Hannachi, Neila Belguith, Ridha M’rad

**Affiliations:** 1 Department of Congenital and Hereditary Diseases, Charles Nicolle Hospital, University Tunis El Manar, 1006 Tunis, Tunisia; 2 Laboratory of Human Genetics LR99ES10, Faculty of Medicine of Tunis, University Tunis El Manar, 1006 Tunis, Tunisia; 3 Department of Radiation Oncology, Salah Azaiez Institute, University Tunis El Manar, 1006 Tunis, Tunisia; 4 Institute of Applied Biological Sciences of Tunis, University Tunis El Manar, Tunis, Tunisia; 5 Laboratory of Pathology Anatomy and Cytology, Tunis, Tunisia

**Keywords:** Li–Fraumeni syndrome, *TP53* germline variants, Breast cancer, Phyllode tumor, Intralobular breast carcinoma, Osteosarcoma, Genotype–phenotype correlation

## Abstract

**Background:**

Li–Fraumeni syndrome (LFS) is a rare autosomal hereditary predisposition to multiples cancers, mainly affecting young individuals. It is characterized by a broad tumor spectrum. To our best knowledge, only one Tunisian study with a confirmed LFS was published.

**Methods:**

Our study focused on the clinical, histopathological and genetic results of two patients with rare tumor phenotype and tried to establish genotype–phenotype correlation. The clinical diagnosis was based on Chompret-Bonaiti criteria relative to LFS. Molecular study was assessed using Sanger sequencing of the hotspot germline variants of *TP53* gene.

**Results:**

We report 2 Tunisian families fulfilling the clinical criteria of Chompret-Bonaiti. The tumor phenotype was bilateral breast cancer (BC) in 27-year-old woman and multiple tumors for the second proband, with an onset age of 14, 35 and 36 yo for osteosarcoma, BC and esophageal cancer respectively. Each of them had a rare histological type of breast cancer associated with LFS, phyllode tumor and intralobular carcinoma. Both patients had cancer family history. The molecular study showed deleterious heterozygous germline *TP53* variants in each index case: The first had a well-known hotspot missense variation c.742C>T p.(R248W) with a rare histological association, explaining genotype phenotype correlation. The second case had a nonsense variation c.159G>A p.(W53*), rare worldwide, extending the phenotype spectrum in LFS. Immunohistochemistry study in tumor samples confirmed the lack of p53 protein expression.

**Conclusions:**

Conclusively, germline *TP53* testing is primordial in patients with a family history suggestive of LFS for clinical practice avoiding genotoxic treatments and adapting the surveillance. National database in LFS listing clinical and mutational data is important to set, particularly for variants rarely reported worldwide. Experience from different countries must be integrated to harmonize global protocols for cancer surveillance in LFS.

## Background

Li–Fraumeni syndrome (LFS; OMIM# 151623) is one of the Mendelian forms of cancer with an autosomal dominant transmission. Since its first description in 1969 by Frederick Li and Joseph Fraumeni and the identification of germline *TP53* variant in 1990, the criteria and the screening guidelines are continuously updated. It’s characterized by high phenotypic heterogeneity in onset age of cancers and a genetic predisposition to broad tumor spectrum comprising bone and soft-tissue sarcoma, brain tumors, premenopausal breast cancers (BC), adrenocortical tumors, plexus choroid tumors, leukemias and lung cancers [1–4]. LFS results mainly from heterozygous germline variant in *TP53* tumor suppressor gene (OMIM# 191170), with a detection rate varying from 18 to 35% of families suggestive of LFS [5–8]. *TP53* encodes for the transcription factor p53, known as the "guardian of the genome" because of its role in the preservation of DNA integrity. It is implicated in the cell cycle arrest, DNA repair, genomic stability, senescence, cell differentiation, autophagy, angiogenesis and apoptosis [9, 10]. To our best knowledge, one Tunisian family has been published with a confirmed LFS [11]. The aim of our study is to present the clinical and genetic particularities of LFS through two confirmed Tunisian families and set a genotype–phenotype correlation.

## Methods

### Clinical data

The radiation oncology department referred two proband patients from two different families to the oncogenetic department for clinical suspicion of LFS. For each family, we applied the Chompret-Bonaiti revised criteria elaborated by the French LFS working group and the 2015 updated version [5, 12] and congregated all cancers with its histopathological diagnosis reported in an expanded pedigree.

### Histology analysis

We gathered tumor’s characteristics: histological subtype and grade, lymph node status and vascular invasion. Immunohistochemical staining was carried out with Ready-To-Use (RTU) primary antibodies: estrogen receptor (ER Clone 6F11—RTU Primary reference PA0151 Leica Bond™), progesterone receptor (PR Clone (16)—RTU Primary reference PA0312 Leica Bond™), Human Epidermal growth factor receptor 2 (HER2 Clone CB11—RTU Primary reference PA0983 Leica Bond™), proliferation index Ki67 (Clone MM1—RTU Primary reference PA0118 Leica Bond™), E-cadherin (Clone 36B5—RTU Primary reference PA0387 Leica Bond™) and p53 expression (Clone DO-7—RTU Primary reference PA0057 Leica Bond™). Immunohistochemical analysis was performed in Leica Bond Max automated immunostainer (Leica, Bannockburn, IL). Detection was assessed using Leica Bond-detection kit with the chromogen diaminobenzidine, followed by hematoxylin counterstain. The tumors tissues were examined by the microscope Nikon Eclipse E200. The photos were taken using the Nikon D5300 (18–55 VR II Kit) Digital camera.

### Mutation screening of *TP53* gene by direct sequencing

An informed consent was signed before genomic DNA extraction on blood samples. Designed primers in *TP53* gene’s exons with adjacent splice sites, under specific PCR conditions, to screen the hotspot variants according to the IARC *TP53* database were used as previously described [13]. BigDye terminator chemistry was used to process sequence reaction (Applied Biosystems, Germany). Variant detection was based on Sanger sequencing (ABI 3130 sequencer Applied Biosystems, Foster City, CA) of PCR products derived [13]. The reference wild-type *TP53* sequence was NM_000546.5, corresponding NP_000537.3, to set the variant’s nomenclature*.* The variations were verified in the extensive International Agency for Research on Cancer (IARC) TP53 Mutation Database R20, July 2019 version (https://p53.iarc.fr/TP53GeneVariations.aspx) (https://tp53.isb-cgc.org/), UMD TP53 Mutations Database (https://p53.fr/tp53-database), Human Gene Mutation Database (HGMD, http://www.hgmd.cf.ac.uk/ac/index.php), Leinden Open Variation Database (LOVD, https://www.lovd.nl) and ClinVar (https://www.ncbi.nlm.nih.gov/clinvar)*.* The functional classification of the variation in p53 was provided by the PHANTM classifier (PHenotypic Annotation of *TP53* Mutations) (http://mutantp53.broadinstitute.org/heatMap/login).

## Results

### Clinical data

#### Family 1 (F1)

The index case was diagnosed with a bilateral BC at the age of 27 years old (yo). The Patey’s radical left mastectomy showed a grade I phyllode tumor associated with an invasive ductal carcinoma of 0.3 cm long axis, SBR grade II with negative axillary lymph nodes. Ductal in situ carcinoma was found in the conservative right breast surgery. Immunohistochemistry studies on FFPE (formalin-fixed paraffin-embedded) tissue sections showed HER2 molecular subtype and proliferation index Ki67 at 30% bilaterally. She had radiation therapy combined with chemotherapy and trastuzumab as adjuvant treatment.

**Fig. 1:**
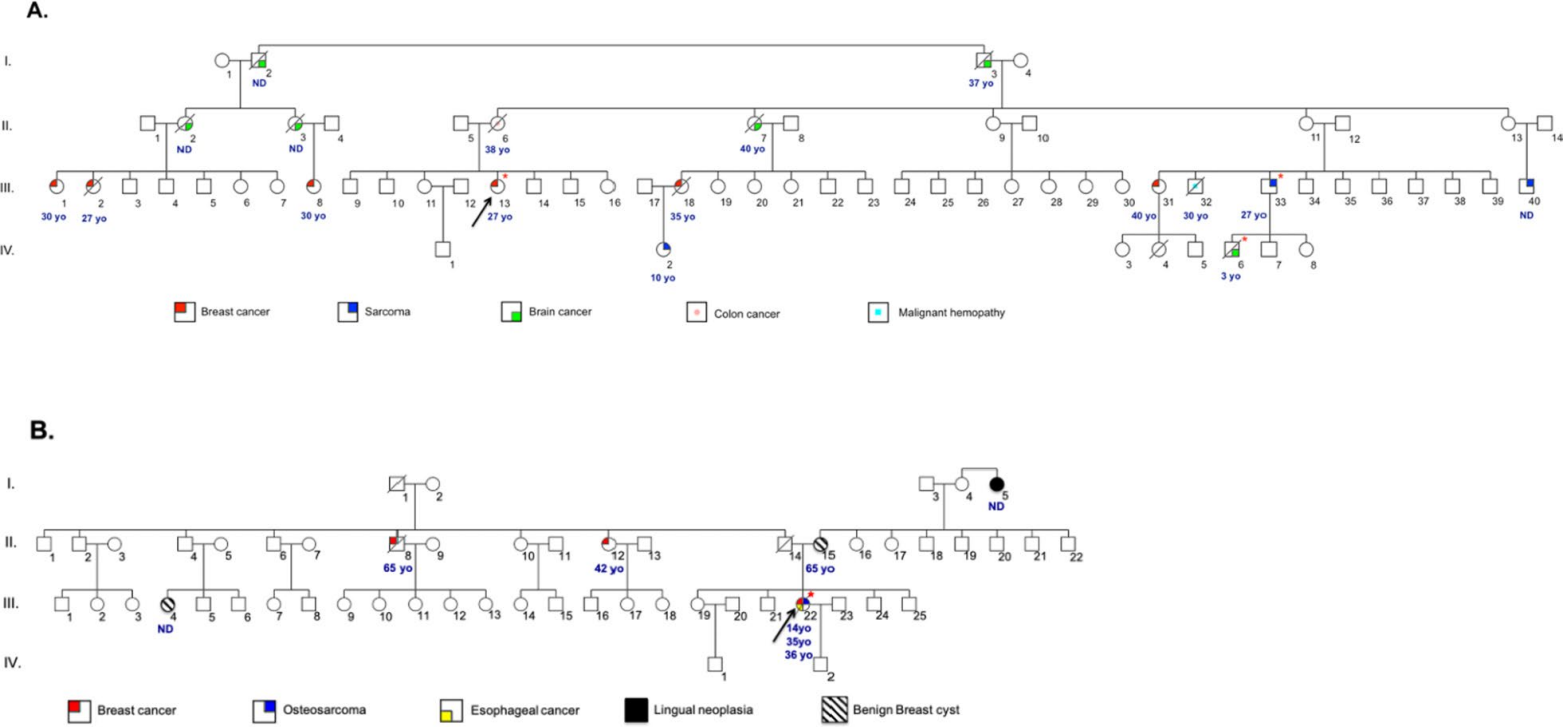
Tunisian families pedigrees. **A**: Family 1 pedigree. **B**: Family 2 pedigree. The arrow indicates the proband case. The age of tumor onset is mentioned below each affected case. The symbol (*) indicates the individuals carrying the deleterious variant for each family. *ND* not determined. *yo* year old

The genetic survey, one year later, revealed that the proband (III.13) had a familial history of tumors belonging to LFS tumor spectrum (Fig. 1A).

#### Family 2 (F2)

**Fig. 2:**
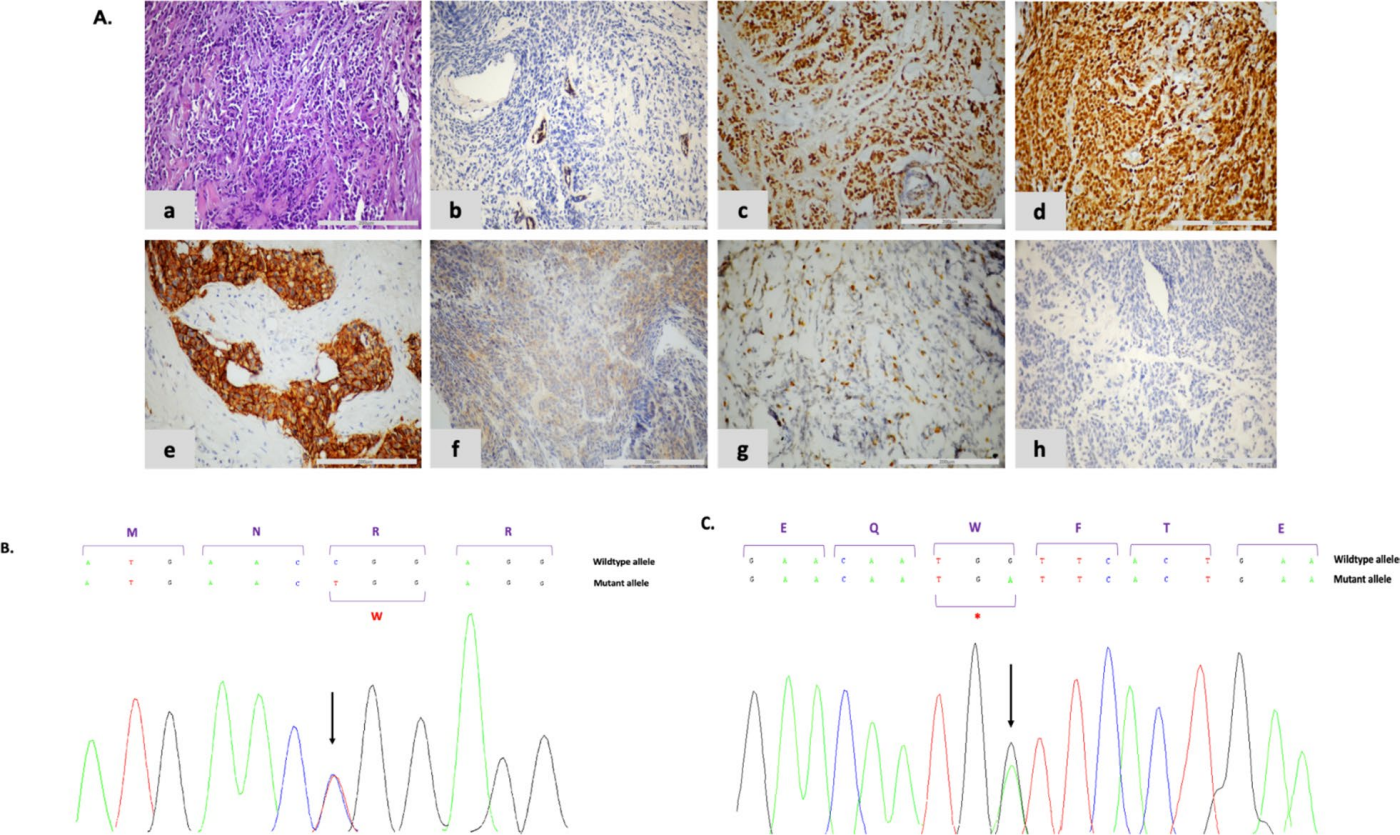
Histological features of breast tumor related to p.(W53*) variant in *TP53* gene and *TP53* electropherograms in both index cases. **A** Immunohistochemical expression patterns (×200 magnification, scale bar corresponds to 200 μm). The tumor sample of F2 index case staining showed a loss of nuclear expression for p53 protein. a: Histopathological examination showing the infiltration of the breast with single files of small, monomorphic and discohesive cells surrounded by a desmoplastic stroma. b: Immunohistochemical staining of E-cadherin showing the negativity of the neoplastic cells. c: Immunohistochemical staining of ER showing nuclear and intensive staining of the neoplastic cells. d: Immunohistochemical staining of PR showing nuclear and intensive staining of the neoplastic cells. e: Positive internal control of HER2. f: Neoplastic cells are negative for Her2. g: Focal expression of Ki67. h. Loss of nuclear expression for p53 protein. **B** *TP53* electropherogram in family 1 with variant c.742C>T p.(R248W). The red letter indicates the new amino-acid change at position 248. **C** *TP53* electropherogram in family 2 with variant c.159G>A p.(W53*). The red symbol (*) indicates the premature stop codon at position 53. **B** and **C** The black arrow shows the variation at the heterozygous state in the reference sequence NM_000546.5 of *TP53* gene. The purple letters refer to the amino-acid sequence. ER: estrogen receptor; HER2: human epidermal growth factor receptor 2; PR: progesterone receptor

The proband had multiple primary tumors, two of which belong to LFS tumor spectrum and first of which occurred before age 46 yo (Fig. 1B). She was followed since the age of 14 yo for an osteosarcoma of the right lower limb for which she underwent surgery followed by adjuvant radiochemotherapy. Lobular carcinoma in the right breast was diagnosed at the age of 35. Immunohistochemical study showed loss of E-cadherin expression in neoplastic cells and positive expression of hormonal receptors (Fig. 2A). She was treated by neoadjuvant chemotherapy followed by radical mastectomy and adjuvant radiochemotherapy. Postoperative follow-up revealed 9 months later an esophageal differentiated epidermoid carcinoma with a circumferential margin less than 1 mm, for which she underwent subtotal esophagectomy.

Family history found besides multiple tumors in proband, two relatives with malignant BC (Fig. 1B).

### Genetic analysis and confrontation to histological data

DNA sequencing in F1 index case revealed heterozygous missense germline variant NM_000546.5: c.742C>T replacing an arginine for a tryptophan at codon 248 p.(R248W) (Fig. 2B). Her relatives (III.33 and IV.6) carried the same variant.

For F2 proband, a deleterious variant c.159G>A p.(W53*) has been identified at heterozygous state, leading to a truncated p53 protein with 53 amino acids, possibly destabilizing its protein interaction (Fig. 2C). Nonsense-mediated mRNA decay (NMD) is likely to occur. We supplemented with a study of p53 expression which showed a lack of protein expression (Fig. 2A-h).

## Discussion

We report two confirmed Tunisian families fulfilling both 2009 and 2015-updated versions of Chompret-Bonaiti criteria with an early bilateral BC in F1 (27 yo) related to p.(R248W) and multiple tumors with an onset age of 14, 35 and 36 yo for osteosarcoma, BC and esophageal cancer respectively for the second proband of F2 due to p.(W53*) (Figs. 1 and 2).

*TP53* gene, located in chromosome 17p13.1, is composed of 11 exons, ten of which are coding for a 393 amino acids (AA) protein p53, composed of five functional domains: two N-termini transactivation domains (1–42 AA and 43–62 AA), a DNA-binding domain (102–292 AA) and two C-termini domains, an oligomerization domain (323–356 AA) and a regulatory domain (363–393 AA) [14, 15]. The central DNA-binding domain, from exons 5–8, involves the hotspot variants (IARC database). About 70% of variants are missense (International Agency for Research on Cancer, IARC TP53 Database, http://www-p53.iarc.fr/).

The p.(R248W), is the second most frequent variation. The tumor pattern of germline p.(R248W) (N = 111) involves BC (29,7%), brain cancer (24,3%), adrenal gland tumors (7,2%), bones and soft tissues tumors (6,3%/6,3%), gastric cancer (3,6%), skin and lung cancers (2,7%/2,7%), biliary tract and colon cancers (1,8%/1,8%), and less than 1% in uterus, rectum, pancreas and liver cancers (IARC database). Our proband F1 had an early-onset bilateral BC (< 31yo), the most frequent cancer type in LFS (31.5%, n = 2591 IARC database) and in p.(R248W). Most of BC in LFS were HER2 positive as F1 index case [5]. We have to highlight that we report a rare histology association of grade I phyllode tumor and an invasive ductal breast carcinoma. Few studies associated *TP53* germline variant with phyllode tumor (Table 1) [16–18]. Detection of this variant in her relatives is an additional argument in favor of the variant’s segregation with the disease and the variant’s phenotype severity.

**Table 1:** Germline *TP53* variants associated with phyllode tumors or lobular carcinoma reported in literature

Histological finding	References	Cohort	*TP53* variants	Tumor onset age (yo)	Tumor phenotype
Phyllode tumors	Mazoyer et al*.* [16]	1/2	c.770T>A p.(L257Q)	11	Osteosarcoma
				15	Phyllode tumor
				22	Soft-tissue sarcoma
	Nogales et al*.* [17]	1	c.1006G>T p.(E336*)	10	Telangiectatic osteosarcoma
				18	Bilateral phyllode tumor
				21	Reticulohistiocytoma
				23	High-grade intraductal carcinoma of the left breast
				23	Multifocal intrafollicular granulosa cell tumor of the right ovary
				24	Retroperitoneum liposarcoma
				25	Leiomyosarcoma
	Prochazkova et al*.* [18]	1	c.742C>T p.(R248W)	27	Malignant phyllode tumor
				17	Malignant fibrous histiocytoma
	Our study: F1 index case	1/2	c.742C>T p.(R248W)	27	Benign phyllode tumor and invasive ductal breast carcinoma
ILC	Rath et al. [19]	*1/213*	c.1000G>C p.(G334R)	44	MDLC
	Meiss et al*.* [20]	1/612	c.725G>A p.(C242Y)	66	Grade 2 ILC ER+/PR−/HER2−
	Petridis et al*.* [21]	0/1434	NA	NA	NA
	Ditchi et al*.* [22]	1/3469	*	*	ILC
	Le AN et al*.* [23]	3/94	*	*	MDLC
	Kuba et al*.* [24]	1/27	*	*	MDLC
	Our study: F2 index case	1/2	c.159G>A p.(W53*)	35	Unilateral ILC ER+/PR+/HER2−

ER: estrogen receptor; HER2: human epidermal growth factor receptor 2; IDC: invasive ductal breast carcinoma; ILC: invasive lobular breast carcinoma;

MDLC: mixed ductal and lobular carcinoma; NA: not applied; PR: progesterone receptor; (*): not determined; (+): positive expression; (−): negative expression

In F2, the deleterious germline variant p.(W53*) affects the N-terminal transactivation domain. This nonsense variation was not referenced in online IARC *TP53* database as germline variant. It was only reported in one child with adrenocortical carcinoma [5] while our patient F2 had multiple tumors, osteosarcoma, lobular breast carcinoma and esophageal epidermoid carcinoma expanding the clinical phenotype. The p.(W53*) has been reported in somatic variations (N = 19) in less than 1% in each site, cited in decreasing order of frequency: vulva, head and neck, oropharynx, ovary, brain, colon and lung cancers (IARC database).

Here again, we report a rare histology association of lobular breast carcinoma and LFS which was reported in very few studies (Table 1) [19–24].

Multiple primary tumors (n = 2 to 6) were reported in 43% of cases (139/322) and were metachronous (83%, 116/139) with a mean gap between the first and the second tumor of 10,7 years (2–26) after radiotherapy [5]. The role of p53 in response to DNA damage could explain the high risk of radio and chemo-induced tumor [9, 10]. The LFS was confirmed late after the third cancer in F2 index case. The esophageal epidermoid carcinoma is probably radio induced.

We underline the interest of addressing patients to oncogenetic consultation as soon as possible, to confirm the LFS early. An appropriate genetic counselling to these families could be given with regular monitoring according to recent recommendations among genetic carriers and personalized care in case of cancers by avoiding genotoxic radiotherapy and chemotherapy [25].

It is insufficient to set a genotype phenotype correlation in the germline variant p.(W53*), since it was only reported in two cases. So the functional impact of these variants would be helpful. The p.(R248W) has a strong dominant negative (DNE) property and a drastically altered transcriptional activity of 0,09% showing a non-functional impact (PHANTM classifier) which could explain the phenotype severity [26, 27]. Furthermore, this variant is one of the major gain-of-function (GOF) variant suggested by mouse models of LFS with interaction of GOF mutants p53 with multiple proteins, such as the nuclease Mre1 leading to inhibit the interaction of MRN complex to DNA double-stranded breaks and induce genetic instability by inactivating ATM protein, contributing to drive and promote tumorigenesis [28, 29]. The p.(W53*) leads to a loss of function effect. Initially, DNE property was not related to the early onset of tumors but correlated the occurrence in type of cancer [26]. In fact, DNE-variants carriers in *TP53* show twice more bone sarcomas and BC [26], which is consistent with F1. Carriers of DNE variant are reported to have severe clinical phenotype with an early onset tumor than those carrying loss of function variant (mean age: 21.3 vs. 27 years) with a statistically significant difference (*p* = 0.0042) [5]. This was not consistent with age of tumor onset in our patients, F1 at 27 yo with a DNE variant and F2 at 14 yo with loss of function variation. DNE variants are observed in 40% of children with osteosarcoma [5], while F2 proband had loss of function variation. Loss of function are known to have less severe phenotype with later tumor onset [26, 27]. These previous arguments could not conclude about the clinical severity of p.(W53*) in F2 proband, but we underline and should be aware of the possibility of childhood cancer in this case, ACC already reported and osteosarcoma in F2 [5].

The penetrance in germline *TP53* variants was variable regarding the variation type and the sex. The highest penetrance was assigned to carriers of p.(R248W) with 58% at 30 years and about 85% before 70 years old [30]. Penetrance is higher in men who tend to develop more tumors especially brain neoplasia and sarcomas before age 30 [30]. In F1, six members were diagnosed with brain tumors including three men, the youngest of which was a three-year-old boy. Contrasting with p.(W53*) which seems to have lower penetrance. The penetrance may be explained by the loss of transcriptional activity [26].

Thus, in routine practice, gradient of severity could be established depending on the type of variant, its functional classification, its penetrance and the early onset of tumors. These data are reliable to set the surveillance protocols. In fact, due to the severity of DNE variants, presymptomatic testing and annual total body magnetic resonance imaging are mandatory and recommended since childhood (National Comprehensive Cancer Network). Contrasting with lower severity variants, frequent in adulthood, where presymptomatic testing are reserved to adults and justify annual breast MRI from the age of 20 years (National Comprehensive Cancer Network). Our data is an example of the exception to the rule, to be taken into account with families harboring p.(W53*), predisposing to early bone cancer and ACC, for whom we propose presymptomatic testing to children and whole body MRI. The first limitation of this study was that we have tested only the proband in family F2 and should investigate the affected members. The second limitation is that we only did a germline sequencing of probands. Reviewing the literature about tumor sequencing in LFS found only some case reports. Sugawara et al. reported a LFS case with p.(R273H) variant associated with an aggressive phenotype, two rhabdomyosarcomas at the ages of 18 months and 21 years [31]. An array-based analysis revealed in the second tumor an amplicon at 5q11.2 including *MAP3K1* gene and the second one at 11q22.2 containing genes: *YAP1* and *BIRC2/3*, known for its anti-apoptosis function, allowing a comprehensive view of cancer progression [31]. An Italian study tried to find genetic modifiers that accelerate tumor development in LFS families other than *MDM2* promoter polymorphisms SNP309T>G (rs2279744) or shortness of telomere length by performing whole-exome analysis (WES) of the trio [32, 33]. The Italian proband developed at 4 year-old, an adrenocortical carcinoma. His mother had an in-situ carcinoma of the right breast at the age of 37 and his father was healthy. He inherited from his mother *TP53*: c.266_269del, p.(S90fs) related to LFS and nonsense variation c.1720C>T, p.(R574*) in ERCC3 from his father, a potential candidate modifier gene that encodes an ATP-dependent DNA helicase and could explain the earlier onset age [32]. Zureick et al. reported a 14-year-old boy with a rare entity of glioblastoma, a gigantocellular-type glioblastoma multiforme, who carried a germline deletion in exons 1 and 2 of *TP53* gene [34]. The family history was made of leiomyosarcoma among his father and grandfather. The proband’s tumoral DNA WES and RNA sequencing showed a homozygous deletion of *PAK1,* a variant p.(R611W) in *TSC2* gene, copy gain of chromosomes 1, 7, 9, 18 and X and copy loss of chromosomes 19 and 22. They highlighted the clinical benefit of precision medicine with a maintain of complete remission after treatment with everolimus during 25 months [34]. These cases provide an insight into targetable pathways that may be involved in LFS cases that we need to study with multicentric projects.

## Conclusions

Tunisian patients adhere to the dichotomous presentation of LFS tumor spectrum with mainly female BC in adults and osteosarcoma in childhood and early adulthood. Our patients showed degrees of phenotypic severity with distinctive types of variants, confirming the heterogeneity of LFS in Tunisian patients. The Tunisian experience allowed us to extend the phenotype spectrum of LFS. After fifty years of the identification of LFS, we are continuously changing and adapting the personalized care of germline *TP53* variant's carriers regarding the surveillance protocols of cancers, stratified according to the type of variants.

## Data Availability

All data analyzed in this study are included in this article. The sequence data are available in the NCBI SRA under the accession number PRJNA803730 (https://www.ncbi.nlm.nih.gov//bioproject/803730).

## Abbreviations

AA: Amino acid
BC: Breast cancer
DNE: Dominant-negative effect
ER: Estrogen receptor
HER2: Human epidermal growth factor receptor 2
LFS: Li Fraumeni syndrome
NMD: Nonsense-mediated mRNA decay
PR: Progesterone receptor
